# Urban green spaces and resident health: an empirical analysis from data across 30 provinces in China

**DOI:** 10.3389/fpubh.2024.1425338

**Published:** 2024-05-30

**Authors:** Yan Bi, Ya Wang, Ding Yang, Jialin Mao, Qifeng Wei

**Affiliations:** ^1^Business School, Chengdu University of Technology, Chengdu, Sichuan, China; ^2^Humanities and Law School, Chengdu University of Technology, Chengdu, Sichuan, China

**Keywords:** urban green spaces, resident health, air quality, social connectivity, urban planning

## Abstract

**Background:**

This study aims to explore the correlation between urban green space coverage and resident health, and to analyze its underlying mechanisms.

**Methods:**

Using panel data from 30 provinces in China from 2006 to 2022, which mainly includes urban green space coverage, general health of the population, air quality, and social connectivity. This research constructed a fixed effects model to perform baseline regression analysis. A series of robustness tests, including variable substitution, controlling for geographical differences, regional robustness tests, and shortening the time span of the study, further verified the robustness of the results. Additionally, mechanism tests were conducted to examine the positive impacts of urban green spaces on resident health by improving air quality and enhancing social connectivity.

**Results:**

The findings indicate a significant positive correlation between urban green space coverage and resident health levels. That is, the greater the area covered with urban green space, the healthier the residents of the area will be. Robustness tests support the reliability of this finding, while mechanism analysis reveals that urban green spaces have a positive impact on the health of the population by improving air quality and increasing social connectivity.

**Discussion:**

This study underscores the importance of urban green space planning in improving resident health and quality of life, providing urban planners with scientific evidence to optimize urban green systems for broader health objectives.

## 1 Introduction

With the acceleration of China’s urbanization and the continuing growth of urban population, the cities are facing increasingly severe environmental and health challenges. As an important part of the urban ecosystem, urban green space has a significant impact on the health status and quality of life of residents. Currently, the planning and construction of urban green space in China have received unprecedented attention. As an important part of the urban ecosystem, urban green space plays a key role in improving air quality, reducing noise, and beautifying the environment. The government is committed to improving the accessibility and availability of green spaces to alleviate resident health problems. The Opinions on Promoting the Green Development of Urban and Rural Construction explicitly proposes the construction of a coordination mechanism for the green development of regions and urban agglomerations, emphasizing the importance of urban ecosystem improvement. The Guidelines on Planning and Land Policies in Support of Urban Renewal (Version 2023) issued by the General Office of the Ministry of Natural Resources (MNR) of China aims to enhance the level of urban planning, construction, and governance to create livable, resilient, and smart cities through the deep integration of planning and land policies. The Urban Green Space Planning Standards issued by the Ministry of Housing and Urban–Rural Development provide national standards and guidance for urban green space planning and promote the standardization and scientification of urban landscaping and greening construction.

Existing studies have mostly relied on questionnaire surveys and micro-empirical studies when exploring the relationship between urban green space environment and residents’ mental health (1). Although these research methods reveal, to a certain extent, residents’ perception and use of green space and the specific impact of green space environment on mental health, they are limited by the size of the sample and geography, geographical limitations, and it is difficult to comprehensively reveal the overall correlation and deep logic between green space and resident health. As a key indicator of urban greening level, the impact of urban green space coverage on resident health has become one of the hotspots of academic research. This study aims to collect and analyze provincial data from a macro perspective, and deeply analyze the impact of urban green space coverage on resident health through empirical evidence. Using large sample data and empirical statistical analysis, the study systematically explores the specific impact of urban green space coverage on resident health and deeply analyses the potential mechanisms behind it, which not only provides new perspectives for understanding how urban green space promotes social interactions and health, but also helps the government and urban planners to make more scientific decisions when formulating relevant policies and plans, which has important theoretical and practical values.

This study explicitly sets the following two research objectives: first, to construct a fixed-effects model to empirically test the positive correlation between urban green space coverage and resident health level using panel data from 30 provinces in China; and second, to further explore how urban green space positively influences resident health through mediating variables such as improving air quality and strengthening social ties. Through this study, we expect to gain an in-depth understanding of the role of urban green space in enhancing resident health, provide a scientific basis for urban planners, and help build a more livable and healthy urban environment. In order to realize the above research objectives, the following research questions are posed in this study: How does urban green space coverage affect the health level of residents? Is this effect realized through mediating variables such as improved air quality and stronger social ties? An empirical analysis was conducted based on data from 30 provinces in China to quantify the positive impact of urban green space on population health.

The first part of this study reviews the relevant research results on the relationship between urban green space coverage and resident health at home and abroad, summarizes the main findings and perceptions of existing studies, and provides a theoretical framework and research ideas for this study. The second part conducts theoretical analyses and puts forward research hypotheses based on the literature review. The third part of the study describes the theoretical model and methodology used in the study, as well as a detailed description of the indicators used, including the measurement of variables, such as urban green space and the health of the population, to ensure the scientific validity and reliability of the study. The fourth part of the empirical results analysis, using empirical data from 30 provinces in China, to explore the specific impact mechanism of urban green space coverage on resident health. This section provides an in-depth analysis of the correlation and degree of influence between urban green spaces and resident health by conducting benchmark regressions, robustness tests and mechanistic analyses. The fifth section, Conclusions and Implications, summarizes the results of the study and puts forward corresponding policy recommendations and practical implications, to provide a scientific basis and practical guidance for urban green space planning, construction, and management, and promote the healthy development of the city and the improvement of residents’ quality of life.

The purpose of this study is to provide a scientific basis for government policymakers and urban planners to promote the rational planning and construction of urban green space. An in-depth study of the relationship between urban green space coverage and resident health, reveals the exact mechanism of the influence of urban green space on resident health and provides specific guidance and strategic recommendations for the design, layout, and management of urban green space. This study is expected to provide important theoretical and practical references for promoting the sustainable development and optimization of urban green spaces around the world, and to make substantial contributions to the creation of healthy and livable urban environments, and to the improvement of residents’ quality of life and sense of well-being.

## 2 Literature review

Urban green space, as the core element of urban ecology, plays a pivotal role in optimizing the layout of the urban environment and improving the quality of life of residents, and is an important guarantee for the sustainable development of the city (2). The impact of urban green space on the health of residents is a multidimensional issue that involves many fields such as environmental science, urban planning, psychology and resident health (3). Many studies at home and abroad have explored the relationship between urban green space and resident health. Many studies at home and abroad have explored the relationship between urban green space and resident health. Green spaces have always been considered an important part of urban ecosystems (4). They not only provide residents with opportunities to access nature, but also help to relieve the stress of urban life and have a positive impact on residents’ psychological state (5). The coverage of urban green space has positive benefits on social health (6). The coverage of urban green spaces has a positive impact on the health of the community.

In recent years, researchers have begun to utilize geographic information systems (GIS) (7) and remote sensing to assess the spatial distribution of urban green spaces and their potential impact on the health of residents, thereby quantifying the degree of spatial coupling between the cooling benefits of green spaces and the distribution of temperature and population. The results found that there is spatial consistency between the cooling benefits of green space and the needs of human settlements. Parks near lakes with higher cooling potential and parks with higher forest cover attracted more slow and long-distance running activities, thus synergistically mitigating the unfavorable thermal environmental effects of the city in summer. Parks with higher cooling potential and high wooded parks are more attractive to slow-distance running activities (8), and synergistically play a certain role in mitigating the unfavorable urban thermal environment in summer (9). The accessibility of green spaces is a guarantee. The accessibility of green spaces provides a useful reference for the development of sheltered housing and helps optimize the spatial layout of the city (10).

Green spaces can be measured more accurately using these techniques and can be analyzed at larger spatial scales. In addition to this, some scholars have also used meta-analysis to systematically review the literature in the database, summarized the significant effect size associations of urban green space with seven aspects of mortality, autonomic activity, endocrine system, immune system, mental stress, emotional potency, and cognitive ability, and summarized multiple potential spatial pathways of action for the health impacts of green space (11).

## 3 Theoretical analysis and research hypothesis

### 3.1 Potential links between urban green spaces and resident health

As an important part of urban ecosystems, the positive effects of urban green spaces on the health of residents have received widespread attention. Research at the intersection of ecology, environmental science and resident health has revealed the multiple ecological services of urban green space in providing recreational space, improving microclimate, reducing heat island effect, and so on (12). These functions not only enhance the ecological quality of cities, but also improve the ecological quality of cities. These functions not only enhance the quality of the urban ecological environment, but also have a significant positive impact on the physical and mental health of residents. In this context, the relationship between urban green space coverage and resident health has become a topic worthy of in-depth discussion. Recent studies have emphasized the role of urban green space in promoting residents’ mental health, pointing out that green space can reduce environmental stress and improve the quality of life (13). The findings of this paper are based on the following hypotheses. Based on these findings, this paper proposes the following hypothesis:

*H*1: There is a positive correlation between the increase in urban green space coverage and the increase in resident health, i.e., the more green space there is in the city, the better the health of the residents.

### 3.2 Mediating role of air quality

Theories from environmental epidemiology and air quality provide possible mechanisms by which urban green spaces can indirectly affect the health of residents by improving air quality (14). Urban green spaces effectively improve air quality by absorbing pollutants from the atmosphere, increasing the negative oxygen ion content of the air, and maintaining soil ecology (15). This process is essential for reducing health problems caused by air pollution. Studies have shown that a good air quality environment can significantly improve the overall health of residents (16). Therefore, this paper further explores how urban green spaces can indirectly contribute to the enhancement of resident health through the mediating variable of improving air quality. Accordingly, the following hypotheses are proposed:

*H*2: Urban green space has a positive impact on resident health through the mediating variable of improved air quality. Specifically, an increase in urban green space coverage can effectively improve air quality, which in turn promotes the improvement of resident health.

### 3.3 The mediating role of residents’ social connectedness

Theories from social psychology and the sociology of health emphasize the importance of social connectedness to the health of the population (17). Urban green spaces, as public places for socializing and leisure, not only promote communication and interaction among residents, but also enhance community cohesion and sense of belonging (18). In addition, the natural environment provided by urban green spaces can bring residents positive emotional experiences and a relaxing leisure environment, which can help to enhance their sense of well-being (19). The social and psychological dimensions of these positives are also important for community well-being. They can alleviate psychological stress, prevent mental health problems, and have a positive impact on the overall health of residents (20). Based on these theories, this paper explores how urban green spaces can indirectly promote resident health by enhancing their social connectedness and well-being. Therefore, the following hypotheses are proposed:

*H*3: Urban green space not only improves the ecological environment, but also indirectly affects resident health by enhancing their social connectedness and sense of well-being. Therefore, as the coverage of urban green space increases, the social well-being of residents will be enhanced, which in turn promotes the improvement of resident health.

## 4 Study design

### 4.1 Model setting

To eliminate unobservable differences between individuals in different provinces as well as to control for time variation, the research model in this paper is a double fixed effects model, as shown in Equation (1).


(1) $$jmjk_{\mathrm{it}}=\alpha_{1}+\alpha_{2}csld_{\mathrm{it}}+\alpha_{3}X_{\mathrm{it}}+\lambda_{i}+\mu_{t}+\varepsilon_{\mathrm{it}}$$

where *i* denotes province and *t* denotes year, the explanatory variables are $jmjk_{\mathrm{it}}$ represents the health level of the population in year *t* of the *i*th province, the core explanatory variables are $csld_{\mathrm{it}}$ represents the urban green space in year *t* of the *i*th province, X denotes the set of control variables, which are geographical attributes—*dysx*, regional healthcare level—*ylsp.,* regional aging level—*llhcd*, regional economic level—*jjsp.,* regional industrial structure—*cyjg*, urban physical activity—*ydsp.,* and $\lambda_{i}$ denotes area fixed effects, the $\mu_{t}$ denotes time fixed effects, and $\varepsilon_{\mathrm{it}}$ is the random error term.

### 4.2 Variable setting and descriptive statistics

#### 4.2.1 Core explanatory variables

**Figure 1:**
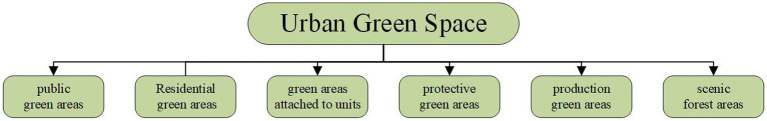
Schematic diagram of the components of the urban green space indicator.

Urban Green Space: This paper refers to the study of Richardson and Elizabeth (21) adopts the green coverage area of the built-up area to express its urban green space coverage. The green coverage area of built-up areas is mainly composed of six parts as shown in the figure below, reflecting the scale and size of urban green space in a region. This paper measures in hectares, and the more this indicator is, the more urban green areas are covered (Figure 1).

#### 4.2.2 Explained variables

Resident health: This paper uses per capita health costs to measure the health level of its residents, which will lead to an increase in per capita health costs when residents have a heavy burden of disease and insufficient preventive health care. This is calculated as shown in Equation (2).


(2) $$\mathrm{Per}\;\mathrm{capita}\;\mathrm{health}\;\cos t_{\mathrm{it}}=\frac{\mathrm{total}\;\mathrm{health}\;\cos t_{\mathrm{it}}}{\mathrm{total}\;\mathrm{populatio}n_{\mathrm{it}}}\text{.}$$

Where $\mathrm{Per}\;\mathrm{capita}\;\mathrm{health}\;\cos t_{\mathrm{it}}$ denotes the *per capita* health cost of city *i* in period *t*, the ratio of the total health cost in that year to the average population in that period.

**Figure 2:**
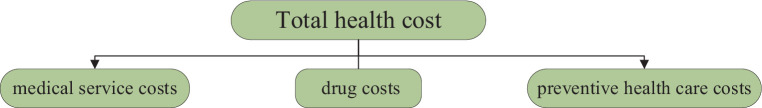
Schematic diagram of total cost components.

The total cost of health has three main components, as shown in Figure 2.

#### 4.2.3 Control variables

Geographical attributes: This paper refers to Zhao’s study (22), considering the large differences in lifestyle between the South and the North, which will have an impact on health. Therefore, this paper assigns a value of 1 to the southern city and 0 to the northern city to distinguish whether the city is located in the southern region of China or the northern region of China. This paper defines “south” based on the Qinling-Huaihe River line as the boundary. This indicator is used to control the influence of living habits, climatic conditions, cultural differences, etc. on the health of residents due to different geographical locations.

Regional level of medical care: This paper refers to Morgan’s study (23), using the number of hospital beds *per capita* in the region to take the natural logarithm to measure the level of healthcare in the city, which is the natural logarithm of the total number of beds provided by all the health institutions within the city (including public hospitals, private hospitals, and community health service centers, etc.).

Level of aging in the area: This paper draws on Hsu’s study (24), and adopts the aging rate of each region to indicate the degree of regional aging, a variable that refers to the proportion of the older adults aged 65 and above to the total population in each province. The aging rate serves as an indicator of urban demographic structure, reflecting the degree of urban population aging. Urban aging may have an impact on resident health, medical needs, social welfare, and other aspects.

Regional gender characteristics: This paper refers to the study of Boerma (25), using the sex ratio by province to reflect regional gender characteristics, which is the ratio of the number of male population to the number of female population in each province, expressed as the number of males per 100 females.

Regional economic level: This paper refers to the study of Sun (26), adopting *per capita* gross regional product (yuan) as an indicator of regional economic level, which refers to the gross regional product divided by the number of resident population in a certain region, reflecting the economic value created *per capita* in the region.

Regional industrial structure: This paper refers to the research of Li Zhongyue (27), adopting the proportion of GDP of the secondary industry as an indicator of regional industrial structure, reflecting the degree of concentration of economic activities and the characteristics of industrial distribution in the region, which refers to the proportion of the gross domestic product of the secondary industry (mainly including the manufacturing industry, the mining industry, and the construction industry, etc.) to the total GDP of the region within a certain region. Different industries may have different impacts on resident health in terms of labor intensity, degree of environmental pollution, and technological innovation capacity.

Urban physical activity: A large number of studies have shown that physical activity has a positive effect on the health of residents (28–30), while the development status of a city’s sports has a positive impact on urban physical activity (31). Therefore, this paper selects the number of ranked athletes to indicate the level of physical activity of urban residents. The number of ranked athletes in a city reflects the sports culture and social atmosphere of the city. When a city attaches importance to sports development and competitive sports, residents will be influenced to actively participate in various forms of sports activities and exercise. Thus, the greater the number of ranked athletes, the higher the level of physical activity in the city. Specific variables are described in Table 1.

**Table 1:** Description of variables and descriptive statistics.

Variable			N	Mean	SD	Min	Max
Type	Name	Symbol					
Explained Variables	Resident health	jmjk	510	3,162	2,217	0	13,968
Explanatory Variable	Urban Green Space Coverage	csld	510	66,750	52,939	2,925	292,976
Control Variable	Geographical Attributes	dysx	510	0.500	0.500	0	1
	Regional Level of Medical Care	ylsp	510	2.817	0.805	0.438	4.320
	Level of Aging in the Area	llhcd	510	0.106	0.0274	0.0547	0.200
	Regional Gender Characteristics	xbtz	510	104.500	3.801	94.920	123.200
	Regional Economic Level	jjsp	510	50,592	31,292	5,787	190,313
	Regional Industrial Structure	cyjg	510	44.130	8.874	15.800	61.500
	Urban physical activity	ydsp	510	1,568	1,252	0	9,678

### 4.3 Data sources

In this paper, a total of 510 research samples from 30 provinces and regions in China from 2006 to 2022 are selected as research objects; Xizang Autonomous Region has been excluded due to severe data deficiency, and the Hong Kong, Macao, and Taiwan regions of China are also not within the scope of this study’s examination due to significant differences in statistical scales compared to other provinces and regions. The data on urban green space coverage was obtained from the CSMAR database, the statistics on resident health were obtained from the *China Health and Wellness Statistical Yearbook*, and the rest of the relevant data was obtained from the *China Statistical Yearbook*, the *China Cultural Relics and Tourism Statistical Yearbook*, and the statistical yearbooks of each city. Data were processed using Stata17.0. Some of the missing data were processed by interpolation.

## 5 Analysis of empirical results

### 5.1 Benchmark regression

In this paper, the POLS method is first used to explore the effect of urban green space on residents’ health, showing that the results are significantly negative, and it is initially concluded that there is a negative correlation between the two. Combined with the results of Hausman test, the *p*-value is 0.000, which is less than 0.005, so this paper is more applicable to the fixed effect model. Therefore, in the baseline regression analysis, this paper chooses the two-way fixed effects model, i.e., considering the fixed effects at the individual and time levels, which helps to reveal the relationship between the variables more accurately. To examine whether there is a positive benefit of urban green space on resident health in urban green space planning, with the help of the model, it is tested that an increase in the area covered by green space in the built-up area is accompanied by a decrease in the *per capita* health costs of residents in the area. The regression results are shown in the table.

Table 2 Column (1) shows the POLS model, and the results indicate that the regression coefficients for the variables *csld* and *jmjk* are −0.004 and significant at the 1% level; Column (2) shows the random effects model, and the results indicate that the regression coefficients for the variables *csld* and *jmjk* are −0.009, and significant at the 1% level; in columns (3) and (4) controlling for the individual fixed effects and the time fixed effects, the regression coefficients of variables *csld* and *jmjk* are −0.011 and − 0.006 respectively, also significant at 1% level; in column (5), both individual and time fixed effects are fixed at the same time, i.e., it is a bidirectional fixed-effects model, and the regression coefficients of variables *csld* and *jmjk* are significantly negative at 1% level. When the urban green space coverage increases by 10%, the related healthcare expenditures of the residents of the area decrease by 0.08%. This set of results indicates that when the area of urban green space coverage increases, the related medical expenditure of the residents of the city decreases, and the baseline regression preliminarily confirms the improvement of the health level of the residents due to the increase of urban green space coverage. Hypothesis 1 is verified (H1).

**Table 2:** Benchmark regression results.

Variables	(1)	(2)	(3)	(4)	(5)
	POLS	RE	FE	Time FE	Two-way FE
csld	−0.004***	−0.009***	−0.011***	−0.006***	−0.008***
	(−3.06)	(−5.20)	(−4.99)	(−3.81)	(−4.27)
ylsp	220.919***	434.605***	722.147***	−325.767**	−1,847.265***
	(3.07)	(3.87)	(4.62)	(−2.25)	(−6.53)
llhcd	6,659.132***	14,867.145***	17,885.246***	3,291.304	3,144.283
	(3.42)	(7.42)	(8.64)	(1.36)	(1.24)
xbtz	5.035	−8.595	−9.854	−13.511	−12.269
	(0.46)	(−0.92)	(−1.06)	(−1.60)	(−1.49)
jjsp	0.052***	0.058***	0.058***	0.044***	0.039***
	(33.52)	(31.58)	(24.84)	(19.98)	(15.55)
cyjg	−78.045***	−61.186***	−47.872***	−24.251***	5.832
	(−16.95)	(−10.41)	(−7.28)	(−3.54)	(0.74)
dysx	−737.664***	−750.037***	−937.851***	−311.736*	−1,863.284***
	(−9.80)	(−4.25)	(−2.68)	(−1.71)	(−5.67)
ydsp	−0.075*	0.009	0.023	0.018	0.007
	(−1.85)	(0.26)	(0.68)	(0.56)	(0.21)
Constant	2,860.846**	1,974.863*	1,099.061	3,347.927***	7,379.788***
	(2.23)	(1.70)	(1.01)	(3.10)	(6.62)
Observations	510	510	510	510	510
R^2^(within)	0.884	0.923	0.924	0.939	0.944
ID FE	NO	NO	Yes	NO	Yes
YEAR FE	NO	NO	NO	Yes	Yes

*t*-statistics in parentheses, ***p < 0.01, **p < 0.05, *p < 0.1.

### 5.2 Robustness tests

#### 5.2.1 Replacement of independent variables

In this paper, we refer to the research of Cawley (32), to conduct the robustness test of replacing the independent variables, replacing the independent variables from the original green coverage area of built-up areas to other similar quantitative indicators - green space area (ha) and green space area of built-up areas (ha). The results of the regression analysis after replacing the independent variables are shown in Table 3, whose coefficients are significantly negative at the 1% confidence level, consistent with the benchmark regression results, implying that the results are statistically reliable. This indicates that the negative correlation between green areas in built-up areas and health costs has not changed. The results of this robustness test enhance the credibility of the study findings and support the finding that increased urban green space coverage may contribute to the improvement of resident health status.

**Table 3:** Robustness tests for replacement of independent variables.

Variables	(1)	(2)
	jmjk	jmjk
ldmj	−0.004***	
	(−3.57)	
jcqldmj		−0.010***
		(−4.80)
Constant	7,930.922***	7,400.134***
	(7.20)	(6.71)
Observations	508	508
R^2^_within	0.944	0.945
Control Variable	Yes	Yes
ID FE	Yes	Yes
YEAR FE	Yes	Yes

*t*-statistics in parentheses, ***p < 0.01, **p < 0.05, *p < 0.1.

#### 5.2.2 Adding dummy variables

In this paper, we refer to the study of Schmidbauer (33) which adds a dummy variable to the original regression model to distinguish between the northern and southern regions. The introduction of this dummy variable aims to control for health differences that may be caused by different geographical locations, such as climatic conditions, dietary habits, and distribution of medical resources. The results of the robustness test show that its coefficient is significantly negative at the 1% confidence level, i.e., after controlling for regional differences between the North and South, the negative relationship between the area of green coverage in built-up areas and *per capita* health costs is still statistically significant, which further strengthens the credibility of the study’s conclusions. The results in Table 4 suggest that the association between increased urban green space coverage and improved Resident health is generalized across geographical regions and that this effect is present in both the northern and southern regions.

**Table 4:** Robustness tests for adding dummy variables.

Variables	(1)
	jmjk
csld	−0.008***
	(−4.27)
Constant	7,379.788***
	(6.62)
Observations	510
R^2^_within	0.944
Control Variable	Yes
ID FE	Yes
YEAR FE	Yes

*t*-statistics in parentheses, ***p < 0.01, **p < 0.05, *p < 0.1.

#### 5.2.3 Sub-sample robustness tests

In this paper, we refer to Wang’s study (34) to conduct regression in sub-samples. Table 5(1)–(2) divides the sample size into two sub-samples, the South and the North, based on geographic regions, to explore whether there is consistency in the impact of urban green space coverage on the health level of residents in different geographic contexts. After regression analyses of the southern and northern subsamples, the results show that in the northern region, when the urban green space coverage increases by 10%, the medical expenditure of the residents in the region decreases by 0.06%; in the southern region, when the urban green space coverage increases by 10%, the medical expenditure of the residents in the region decreases by 0.012%, and the coefficients of the influence of urban green space on the health level of the residents are relatively close in the southern and northern regions, and both are at the 1% confidence level. All of them are significant at the level of 1% confidence interval. It shows that even if the climatic conditions of the north and south are different and the living habits of the residents are not the same, the negative relationship between the built-up area of green coverage and the health level of the residents remains significant, i.e., the geographical differences do not affect the positive effect of the urban green coverage on the health of the residents. Table 5(3)–(4) quantify the sample into two sub-samples of high and low aging, using the average of aging rate in each region as the zero cut-off point. The regression results show that urban green spaces have a significantly negative impact on the health of the population. In particular, in areas with a low degree of aging, when the coverage of urban green space increases by 10%, the related medical expenditure of residents in the area decreases by 0.06%; in areas with a high degree of aging, when the coverage of urban green space increases by 10%, the related medical expenditure of residents in the area decreases by 0.08%. It can be seen that the higher the degree of aging in the region, the greater the impact of urban green space on the health of residents, indicating that the role of urban green space on the health of residents is greater in the older adult group. The results of the categorical robustness test further enhance the reliability of the findings, confirming that the findings apply not only to the overall sample, but also equally to the sub-samples by region and age.

**Table 5:** Sub-sample robustness tests.

Variables	(1)	(2)	(3)	(4)
	South	North	Young	Old
csld	−0.006**	−0.012***	−0.006***	−0.008*
	(−2.45)	(−3.76)	(−3.35)	(−1.82)
Constant		4,519.475**	3,717.443***	8,117.125***
		(2.36)	(3.80)	(3.58)
Observations	255	255	292	218
Number of id	0.959	0.947	0.961	0.930
Control Variable	Yes	Yes	Yes	Yes
ID FE	Yes	Yes	Yes	Yes
YEAR FE	Yes	Yes	Yes	Yes

*t*-statistics in parentheses, ***p < 0.01, **p < 0.05, *p < 0.1.

#### 5.2.4 Reduction in sample study time

In order to reduce the interference of long-term trends and cyclical factors as well as the potential impact of possible policy adjustments, economic conditions, demographics, and other factors over time, this paper refers to the study by Neugebauer (35). The period of the data is reduced from 2006–2022 to 2010–2022 for robustness testing. As the results in Table 6 show, the coefficient remains significantly negative at the 1% confidence level after shortening the period. This suggests that the negative relationship between the green coverage area of built-up areas and *per capita* health costs remains significant even over different time horizons, and that the positive impact of urban green space coverage on resident health is robustly present. In addition, from 2006 to 2020, when the urban green space coverage increased by 10%, the related health expenditure of residents in the area decreased by 0.16%; from 2006 to 2018, when the urban green space coverage increased by 10%, the related health expenditure of residents in the area decreased by 0.12%. At the same time, as shown in the figure, in the context of a series of policies formulated by the government to improve green space, the area of urban green space in recent years has shown a year-on-year trend of increasing, and the overall quantity has improved, indicating that the government’s policies on the construction of urban green space have achieved certain results (36). In addition, with the growth of time, the function of urban green space has been increasingly improved (37). In summary, with the growth of time, the area of urban green space has gradually increased and its function has been gradually improved, which has had a greater impact on the overall health level of residents.

**Table 6:** Robustness test for shortened sample study time.

Variables	(1)	(2)
	2006–2020	2006–2018
csld	−0.009***	−0.007***
	(−4.66)	(−3.42)
Constant	5,240.649***	3,848.532***
	(5.60)	(4.58)
Observations	420	360
R2_within	0.945	0.946
Control Variable	Yes	Yes
ID FE	Yes	Yes
YEAR FE	Yes	Yes

*t*-statistics in parentheses, ***p < 0.01, **p < 0.05, *p < 0.1.

**Figure 3:**
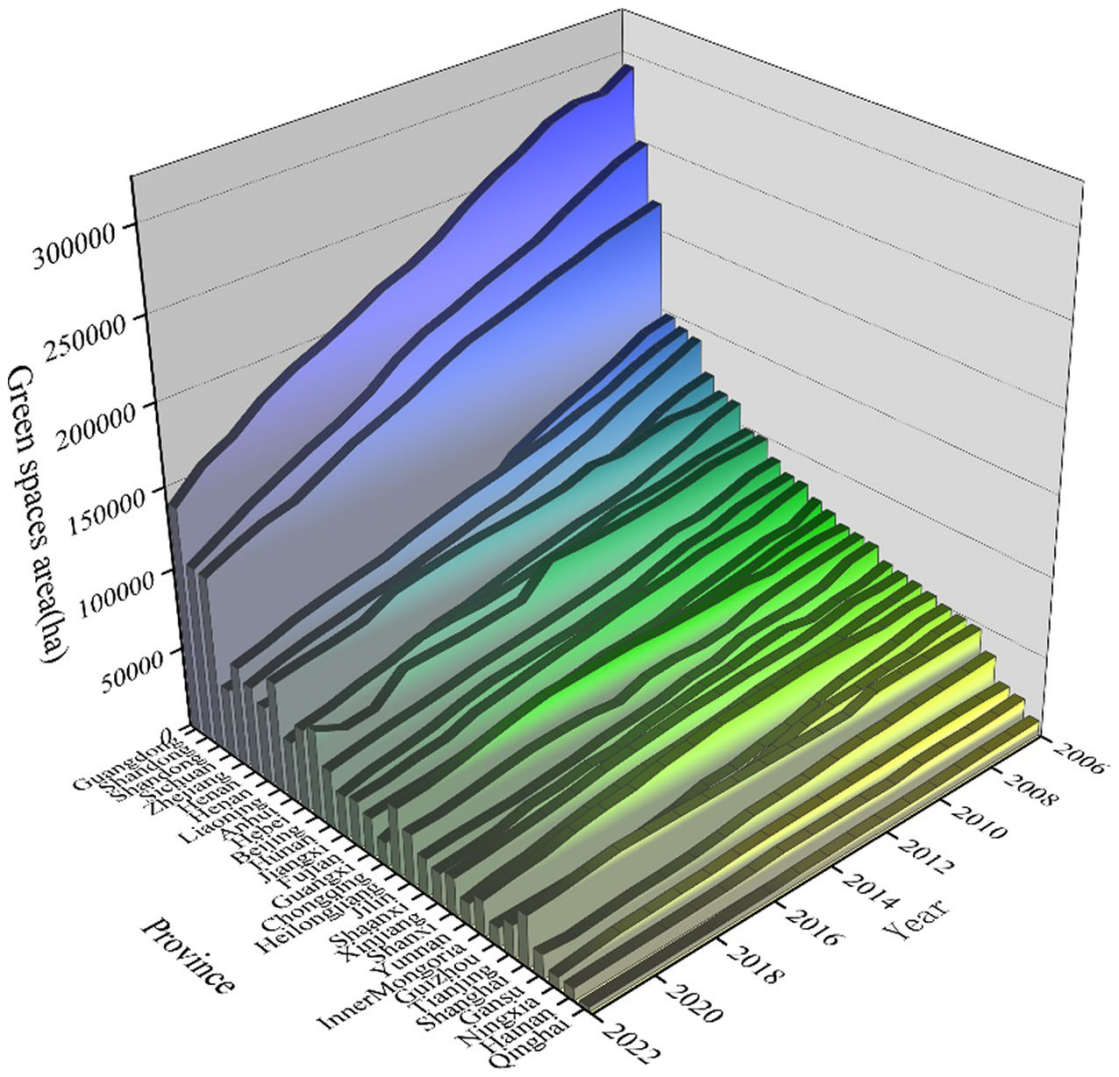
The trend of change in the area covered by urban green space.

The following figure demonstrates the trend of green space coverage provinces from 2006 to 2022 in China. And it can be seen that the overall provinces have shown a year-on-year growth trend, although the base area of green space coverage is not consistent. This positive trend indicates that many of Chinese policies on urban greening and ecological civilization have achieved positive results, and are of great significance in enhancing the quality of life of urban residents, improving the urban ecological environment and promoting sustainable development (38, 39) (Figure 3).

### 5.3 Mechanism testing

The empirical finding that urban green spaces enhance residents’ health has been established. This paper builds upon theoretical analyses to elucidate its mechanistic role, focusing on regional air quality and residents’ social interactions. Referring to Jiang’s study (40), the following model is used to test the mechanism, as shown Equation (3).


(3) $$M_{\mathrm{it}}=\beta_{1}+\beta_{2}csld_{\mathrm{it}}+\beta_{3}X_{\mathrm{it}}+\lambda_{i}+\mu_{t}+\varepsilon_{\mathrm{it}}$$

**Figure 4:**
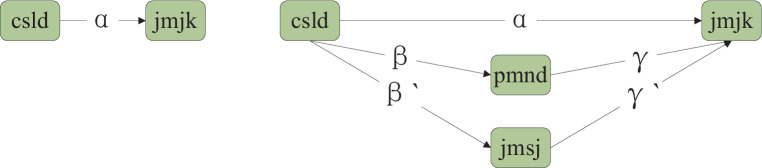
The path analysis diagram of the mediating role.

Where $M_{\mathrm{it}}$ are mechanism variables from two perspectives of regional air quality and residents’ behavior, and the rest of the variables are the same as in the baseline regression model. The mechanism variable for regional control quality is pmnd expressed as the total annual PM2.5 concentration in each province, which refers to Geng’s study (41)and the data are from the CSMAR database; the mechanism variable of the social relevance of residents is *jmsj* is expressed by the number of cultural institutions in each province, where the cultural institutions mainly include the four categories of museums, public libraries, art performance venues, and art performance teams, and the data are from the China Culture and Tourism Statistical Yearbook. The path analysis diagram of the mediating role in this study is shown in Figure 4.

#### 5.3.1 Urban green spaces and regional air quality

Table 7 reports the regression results of the air quality mechanism test. In Column (2), the regression results illustrate the relationship between urban green space and urban air quality. The coefficient is significantly negative at the 1 percent confidence level, suggesting that an increase in urban green space corresponds to a decrease in PM2.5 content, indicating improved air quality in the area. In addition, a large number of studies at home and abroad have shown that air quality has a significant positive impact on the health of residents, the better the regional air quality, the healthier the residents of the region. For example, Geng Guannan studied the long-term trend of PM2.5 exposure and its impact on health in China from 2002 to 2017, and the results showed that higher PM2.5 concentrations, i.e., the worse the air quality, will harm health (41–44). Therefore, it can be concluded that urban green spaces influence the air quality of the area, thereby affecting residents’ health. That is, the more urban green spaces there are, the better the air quality in the area, and the healthier the residents, thus validating Hypothesis 2 (H2).

**Table 7:** Regression results of the mechanism test of urban green space and regional air quality.

Variables	(1)	(2)
	pmnd	pmnd
csld	−0.008***	−24.877***
	(−4.27)	(−4.58)
Constant	7,379.788***	−4664018.809
	(6.62)	(−1.51)
Observations	510	510
R2_within	0.943	0.545
Control Variable	Yes	Yes
ID FE	Yes	Yes
YEAR FE	Yes	Yes

*t*-statistics in parentheses, ***p < 0.01, **p < 0.05, *p < 0.1.

#### 5.3.2 Urban green spaces and socialization of residents

The regression results of the mechanism test of residents’ social connectedness are reported. Column (2) of Table 8 indicates that the effect of urban green spaces on residents’ social connectivity is significantly positive, suggesting that the more urban green spaces there are, the greater the social connectivity among residents in the area. Column (3) reports the effect of residents’ social connectedness on resident health, which is significantly negative at the 1% confidence level, indicating that the more social connectedness residents have in the area, the less healthcare expenses they incur, thus indicating better health. This conclusion is consistent with existing research results (17). Therefore, it can be concluded that urban green space is beneficial to the health of residents by increasing their social connectedness, thus testing hypothesis 3 (H3).

**Table 8:** Regression results of the mechanism test on the association between urban green spaces and the socialization of residents.

Variables	(1)	(2)	(3)
	jmjk	jmsj	jmjk
csld	−0.008***	0.010***	−0.006***
	(−4.27)	(8.95)	(−2.87)
jmsj			−0.231***
			(−2.87)
Constant	7,379.788***	−1,941.245***	6,931.774***
	(6.62)	(−3.02)	(6.20)
Observations	510	510	510
Number of id	30	30	30
Control Variable	Yes	Yes	Yes
ID FE	Yes	Yes	Yes
YEAR FE	Yes	Yes	Yes

*t*-statistics in parentheses, ***p < 0.01, **p < 0.05, *p < 0.1.

## 6 Conclusion and implications

### 6.1 Conclusion

With the acceleration of global urbanization, resident health has become a topic of global concern. Urban green space is a key factor affecting resident health. With the rise of the global concept of sustainable development, the impact of urban green space on resident health has received attention from all walks of life. In this study, empirical analyses were conducted based on relevant data from 30 provinces in China spanning from 2006 to 2022. Employing a double fixed-effects model, we aimed to uncover the significant positive correlation between urban green space and residents’ health levels, while simultaneously exploring the potential mechanisms underlying this relationship. The findings indicate that urban green spaces positively influence resident health through two main pathways: enhancing air quality and fostering social connectedness. These mechanisms collectively contribute to the overall improvement of resident health. In addition, to ensure the robustness of the results, this paper further conducts robustness tests to verify the consistency of the positive effects of urban green space on resident health across different geographic regions and periods, which further supports the idea that urban green space is a key factor influencing resident health levels.

However, although this study provides strong evidence on the relationship between urban green spaces and the health of the population, there are some limitations and potential directions for future research. Firstly, the single-country limitation suggests the need to investigate whether other countries have the same experience. As this study only selected data from China from 2006 to 2022, the results may have some limitations. Future research should be broadened to include more other countries. Secondly, this study mainly focused on the impact of total green space area on health, and future studies could further explore the specific impact of different types of green space (e.g., parks, street trees, community gardens, etc.) on health. In addition, the health of residents is a complex multifactorial issue, and future studies could consider more socio-economic factors, such as education level, income level, and type of occupation, in order to understand more comprehensively the relationship between urban green space and the health of residents. Finally, increasing the complexity of the model, taking into account specific parameters and sophisticated modeling techniques, is essential to understand the intricate interactions between urban planning, green spaces and public health.

This study provides a scientific basis for achieving the strategic goal of “Healthy China” and highlights the importance of green space systems in urban planning all over the world. The study of urban green space can help promote the improvement of the urban ecological environment, enhance the quality of life of residents, promote global sustainable development, and address global challenges such as climate change. We expect that this study will stimulate more attention and action to promote the optimization and upgrading of urban green spaces and contribute to a healthier and more livable urban environment. In conclusion, the study of urban green spaces is of great global significance, and by strengthening the study and protection of urban green spaces, we can work together to build healthier, more livable, and sustainable global cities.

### 6.2 Implications

#### 6.2.1 Policy implications

To enhance the health and quality of life of urban dwellers, governments and urban planners need to take proactive steps to maximize the benefits and functions of urban green spaces. The primary step involves increasing funding for urban green spaces to expand public green areas significantly. Secondly, optimizing the layout of urban green spaces to ensure fairness and accessibility in their distribution, allowing all residents to conveniently enjoy the benefits brought by green spaces. Improving the quality of urban green spaces is critical. This includes diversifying plant species, bolstering the maintenance and management of these areas, and boosting their multifunctional uses. Urban green spaces should serve not only as areas for relaxation and recreation but also as safe havens during emergencies and venues for environmental education. Designing green spaces as multifunctional hubs that integrate leisure, health promotion, and environmental education enhances their utility. Installing walking trails, bicycle paths, and outdoor exercise equipment can transform these areas into active zones that encourage physical activity and heighten health consciousness among residents. Simultaneously, fostering community engagement and involving residents in the planning and management of green spaces not only enhances community cohesion but also promotes the sustainable utilization of these areas. Through these comprehensive measures, urban green spaces will more effectively foster the physical and mental well-being of residents, thereby contributing to the establishment of healthy and livable urban environments.

#### 6.2.2 Theoretical implications

Academic researchers are instrumental in advancing the development and planning of urban green spaces. They should encourage interdisciplinary collaboration among fields such as urban planning, environmental science, resident health, psychology, and economics. This approach deepens the theoretical and practical understanding of urban green space development and supports practical applications. Researchers should strive to merge theoretical insights with empirical studies, providing actionable strategies to policymakers and helping address real-world challenges. Establishing an urban green space impact monitoring and evaluation system is also vital. Furthermore, academic researchers can establish a monitoring and evaluation system for the effectiveness of urban green spaces, regularly assessing their impact on residents’ health and the urban environment, ensuring that urban green spaces meet their intended goals and adapt to emerging challenges.

## Data availability statement

The original contributions presented in the study are included in the article/Supplementary material, further inquiries can be directed to the corresponding author.

## Author contributions

YB: Data curation, Formal analysis, Investigation, Methodology, Software, Writing – original draft. YW: Conceptualization, Funding acquisition, Project administration, Supervision, Writing – review & editing. DY: Data curation, Formal analysis, Investigation, Writing – original draft. JM: Data curation, Formal analysis, Investigation, Writing – original draft. QW: Conceptualization, Project administration, Resources, Supervision, Visualization, Writing – review & editing.
